# Real-World Outcomes of Selective *RET* Inhibitor Selpercatinib in the United States: Descriptive, Retrospective Findings from Two Databases †

**DOI:** 10.3390/cancers16223835

**Published:** 2024-11-15

**Authors:** Chi-Yin Liao, Carmen Gonzalez-Ferrer, Samuel Whipple, Patrick M. Peterson, Scott S. Barker, Naleen Raj Bhandari, Feng Wang

**Affiliations:** Eli Lilly and Company, Indianapolis, IN 46285, USA; chiyin.liao@lilly.com (C.-Y.L.); gonzalez_ferrer_carmen@lilly.com (C.G.-F.); whipple_sam@lilly.com (S.W.); peterson_patrick@lilly.com (P.M.P.); barker_scott_s@lilly.com (S.S.B.); nrbhandari@lilly.com (N.R.B.)

**Keywords:** *RET*, selpercatinib, survival outcomes, real-world effectiveness, lung cancer, thyroid cancer, rearranged during transfection (*RET*) inhibitor

## Abstract

Objectives: This study described real-world patient characteristics and outcomes among selpercatinib-treated patients in the United States, using the Flatiron Health electronic health record-derived deidentified database (FHD) for advanced/metastatic non-small cell lung cancer (a/mNSCLC) and Optum’s de-identified Clinformatics^®^ Data Mart Database (CDM). Methods: Patients initiating selpercatinib treatment between 08MAY2020 and 30JUN2023 were included. We evaluated real-world time to selpercatinib treatment discontinuation or death (rwTTDd) and time to next treatment or death (rwTTNTd) using Kaplan–Meier analyses. Medication possession ratio (MPR) was estimated as a measure of medication adherence in CDM patients. Results: In a/mNSCLC patients from the FHD (N = 68), the median rwTTDd and rwTTNTd were 22.4 [95%CI: 13.3–NR] and 21.0 [95%CI: 11.6–NR] months, respectively. In CDM, these durations were 12.1 [95%CI: 9.6–NR] and 16.2 [95%CI: 9.6–NR] months for lung cancer (n = 43), while these were not reached for thyroid cancer (n = 24) patients. The median MPR was 0.98 [IQR: 0.84–1.00] among all patients in the CDM (N = 75), with 77.3% of patients adhering (MPR ≥ 0.80) to selpercatinib. Conclusions: Real-world outcomes in this older and frailer patient cohort align with phase 3 trial results, further supporting selpercatinib as the standard of care for patients with *RET*-altered cancers. Early testing for the detection of *RET* alterations remains essential.

## Simple Summary

Selpercatinib, a highly selective and potent oral REarranged during Transfection (*RET*) inhibitor, is approved for treating *RET*-altered lung cancer, thyroid cancer, and other solid tumors in the United States. However, there is limited evidence regarding its real-world effectiveness. This study described patient characteristics, treatment patterns, and outcomes among selpercatinib-treated patients in the United States using two real-world data sources. Most patients initiated selpercatinib as a monotherapy. The median real-world time to treatment discontinuation or death and the median real-world time to the next treatment or death ranged from 12.1 to 22.4 months and 16.2 to 21.0 months, respectively, in patients with lung cancer across both databases, while these were not reached for patients with thyroid cancer. Study findings suggest that real-world outcomes associated with selpercatinib use in a cohort of older and frailer patients are largely consistent with the results observed in phase 3 clinical trials.

## 1. Introduction

REarranged during Transfection (*RET*), a gene that encodes a transmembrane receptor tyrosine kinase, was found to be involved in oncogenic signaling and malignancies [1,2]. Genomic alterations in *RET* can abnormally activate the unregulated oncogenic signaling that triggers cell growth and subsequent *RET*-activated cancers [3,4]. The following two primary mechanisms are known: (1) chromosomal rearrangements that fuse the protein kinase domain with other proteins; and (2) point mutations that directly or indirectly activate the protein kinase [5]. Such genomic alterations in *RET* have been identified in a variety of tumor types, including *RET* mutations in medullary thyroid cancer (nearly all for hereditary and 25–50% for sporadic) and *RET* fusions in non-small cell lung cancer (1–2%) and papillary thyroid cancer (10%), and less frequently in other types of tumors [2,6,7,8,9,10,11].

Selpercatinib, a first-in-class, highly selective, and potent *RET* kinase inhibitor with CNS activity, was approved in the United States for the treatment of *RET*-activated, advanced, or metastatic lung and thyroid cancers [12,13]. This approval, granted by the U.S. FDA on 8 May 2020, was based on the efficacy and safety findings of selpercatinib among patients with metastatic *RET* fusion-positive non-small cell lung cancer (NSCLC), and metastatic *RET*-mutant medullary thyroid cancer and metastatic *RET* fusion-positive thyroid cancer from a single-arm, phase 1–2, LIBRETTO-001 trial (NCT03157128) [12,14,15,16,17]. In addition to the LIBRETTO-001 trial, the phase 3 LIBRETTO-431 trial (NCT04194944) showed that first-line selpercatinib significantly extended progression-free survival (PFS) in patients with advanced *RET* fusion-positive NSCLC, compared to platinum-based chemotherapy with or without pembrolizumab (mPFS: 24.8 [17.3–not estimable] vs. 11.2 [8.8–16.8] months; HR: 0.48 [0.33–0.70], *p* < 0.001 in overall intent-to-treat analysis) [18]. The phase 3 LIBRETTO-531 trial (NCT04211337) further established selpercatinib’s efficacy in first-line treatment, showing superior PFS compared to physicians’ choice of cabozantinib and vandetanib in treating patients with advanced *RET*-mutant medullary thyroid cancer (mPFS: not reached vs. 16.8 [12.2–25.1] months; HR: 0.28 [0.16–0.48], *p* < 0.001) [7]. Real-world data describing treatment patterns and outcomes of selpercatinib are limited, particularly those in the United States, to provide supportive evidence of benefit from clinical trials to a real-world setting [19,20,21,22].

Thus, this descriptive study aimed to describe patient characteristics, treatment patterns, and outcomes among cohorts of patients initiating selpercatinib treatment in clinical practice settings.

## 2. Methods

### 2.1. Study Design and Data Sources

This was a retrospective, descriptive, observational study of patients receiving selpercatinib treatment in the US. Flatiron Health’s electronic health record-derived de-identified database (FHD) comprising patients with advanced or metastatic NSCLC (a/mNSCLC) from January 2011 to June 2023 and Optum’s administrative claims de-identified Clinformatics^®^ Data Mart Database (CDM) from November 2019 to June 2023 were used in this study.

The FHD is a longitudinal database containing de-identified patient-level information that has been curated through technology-enabled abstraction and verified by oncology specialists [23,24]. This database encompasses 15% of all U.S. cancer patients, representing a varied and geographically widespread population from around 280 oncology clinics (approximately 800 care sites). Most patients in the database are those who receive medical care in community oncology settings; however, the proportions of patients from community or academic settings in a study may fluctuate based on the derived analytic cohort.

The CDM is a HIPAA-compliant, de-identified data source that compiles administrative health claims data from members of large commercial and Medicare Advantage health plans [25]. The data include patient-level enrollment information and verified adjudicated claims related to all medical and pharmacy healthcare services, providing insights into healthcare costs and resource utilization across a diverse population with more than 65 million unique lives over a 9-year period (1/2007 through 12/2021) in all 50 states.

### 2.2. Study Cohorts and Subgroups

For both the FHD and CDM, patients were included if they initiated selpercatinib between 8 May 2020, and 30 June 2023. The index date was defined as the earliest date of selpercatinib initiation. Any patients with evidence of selpercatinib use prior to its FDA approval date, 8 May 2020, were excluded. Only patients with a positive *RET* fusion biomarker test result up to 30 days after the index date were included from FHD, while this information was not available in CDM. From CDM, patients were included if they had at least two paid prescription claims for selpercatinib on different dates during the index period, at least 180 days (with an allowable gap of 30 days) of continuous enrollment in medical and pharmacy benefits before the index date, and at least 30 days post-index enrollment. The CDM cohort was further categorized into major subgroups by tumor type.

### 2.3. Study Variables

Baseline demographic and clinical characteristics were assessed as available from both FHD and CDM. The baseline period was defined as any time prior to the index date in the FHD and as 180 days prior to the index date in CDM. Treatment patterns included descriptions of immediate pre- and post-index treatment regimens from both databases. For FHD, the available characterization of lines of therapy (LOTs) was used. As for CDM, the pre-index treatment was captured based on treatments received prior to 90 days and post-index treatment was captured based on treatments received in the first 28 days of discontinuation or a change in the index regimen.

Outcomes included real-world time to treatment discontinuation or death (rwTTDd) and real-world time to next treatment or death (rwTTNTd) for both databases. rwTTDd was defined as the time from the index date to the last administration date of selpercatinib before an event occurred. The event included the initiation of a new LOT that did not include the selpercatinib, death, or a ≥90-day gap between the last selpercatinib administration and the last known activity date or the study end date, whichever occurred first. rwTTNTd was calculated as the time from the index date to the start of the next line of treatment that may or may not include selpercatinib or death, whichever occurred first. If none of these conditions were met, patients were censored at the last known activity date or at the end of the study.

An additional outcome included adherence to selpercatinib treatment, measured using the medication possession ratio (MPR) among patients included from CDM [26]. Patients were considered adherent to selpercatinib treatment if their MPR was ≥0.8 (80%)—a threshold commonly used in similar observational studies using a claims database [27,28,29]. MPR was calculated as the sum of days of supply of selpercatinib divided by the total number of days from the index date (inclusive) until the earlier date of treatment discontinuation, disenrollment, or death. For the calculation of the sum of days’ supply, selpercatinib prescriptions dispensed on the same day with different strengths were first combined as one fill up to 320 mg daily, which is the recommended daily dose for those with a body weight of 50 kg or higher [13]. If an early fill overlapped with a previous fill with a different daily dose, the overlapping drug supply was discarded; if it was the same dose, it was assumed that the patient was stockpiling, and the days’ supply was summed. Any inpatient hospitalization periods that began during the at-risk time were not included, and any MPR values that surpassed 1.0 (100%) were truncated at 1.0 (100%).

### 2.4. Statistical Analyses

Descriptive analyses were conducted across both databases to describe baseline characteristics and characteristics of index selpercatinib treatment. Continuous variables were presented as the mean along with the standard deviation (SD) or median along with the interquartile range (IQR), while categorical variables were expressed in terms of frequency and percentages. Sankey diagrams were used to illustrate immediate pre-index, index, and post-index treatment sequences. Time-to-event outcomes were estimated using the Kaplan–Meier method. All statistical analyses were descriptive and conducted using SAS 9.4 and R 4.1.0.

Sensitivity analyses were performed for the time-to-event outcomes and MPR. The first sensitivity analysis was performed for rwTTDd and considered the initiation of a new line of treatment as treatment discontinuation, regardless of whether the new treatment regimen included selpercatinib or not. The second sensitivity analyses considered death as censoring rather than an event to estimate the time to treatment discontinuation (rwTTD) and time to next treatment (rwTTNT). The sensitivity analysis of MPR limited the calculation of MPR to within 180 days.

## 3. Results

### 3.1. Sample

**Figure 1:**
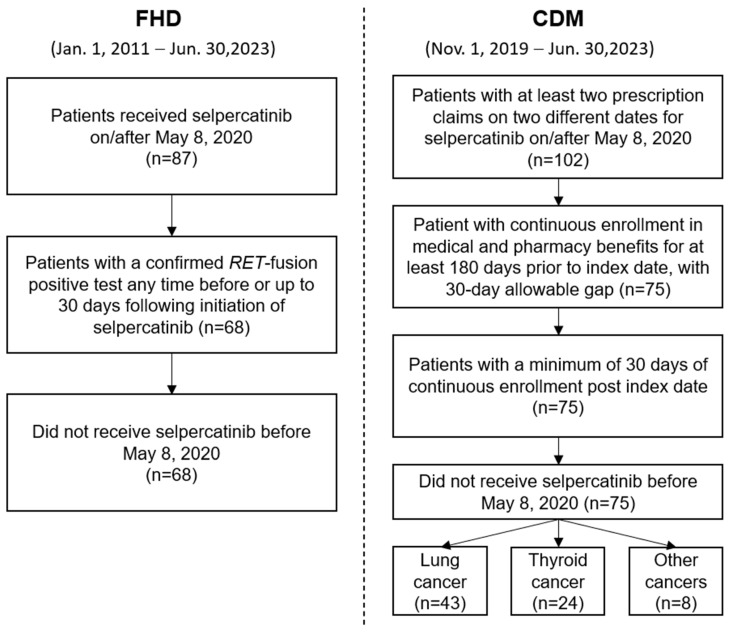
Study cohorts and eligibility criteria.

After eligibility criteria were applied, a total of 68 patients with *RET* fusion-positive a/m NSCLC who initiated selpercatinib were obtained from the FHD, while 75 patients, including those having lung (57.3%, n = 43) or thyroid cancer (32.0%, n = 24), were identified from CDM (Figure 1). The median follow-up time was 13.1 months (IQR: 6.6–26.8) for the FHD cohort, and 8.6 months (IQR: 4.8–16.7), 8.2 months (IQR: 4.5–12.1), and 10.4 months (IQR: 6.6–27.5) for the overall, lung cancer, and thyroid cancer cohorts in CDM, respectively.

### 3.2. Patient Characteristics at Baseline

#### 3.2.1. FHD

Table 1 shows baseline demographics and clinical characteristics at baseline in FHD. Among the FHD cohort, patients were predominantly male (55.9%, n = 38) and white (66.2%, n = 45), with a median age of 67 years (IQR: 60.0–74.0) and an average body weight of 82.5 kg (SD: 23.1). Most were non-smokers (55.9%, n = 38) treated in a community practice setting (63.2%, n = 43), primarily Stage IV (82.4%, n = 56), with non-squamous cell carcinoma histology (98.5%, n = 67) and had ECOG PS of 0/1 at the index date (73.6%, n = 50). Co-occurring biomarker alterations at the initiation of selpercatinib were rare (n ≤ 5 patients had ALK, BRAF, EGFR and/or KRAS mutations), except for PD-L1 positive status (63.2%, n = 43).

#### 3.2.2. CDM

For the CDM cohort, the median age at index date for the overall sample, lung cancer, and thyroid cancer patients was 65.0 (IQR: 55.0–74.0), 67.0 (IQR: 61.0–76.0), and 61.5 (IQR: 47.0–73.5) years, respectively (Table 2). In the overall sample, 54.7% were male, with 44.2% in the lung cancer subgroup (n = 43) and 66.7% in the thyroid cancer subgroup (n = 24). Most of the patients were white, with a Charlson Comorbidity Index score of 1–2 across all groups. Baseline metastasis was present in over half of the patients, with the most common sites being bone/bone marrow and lymph. The lung cancer patients in the CDM show a similar age distribution to the NSCLC patients in the FHD.

### 3.3. Treatment Patterns

**Figure 2:**
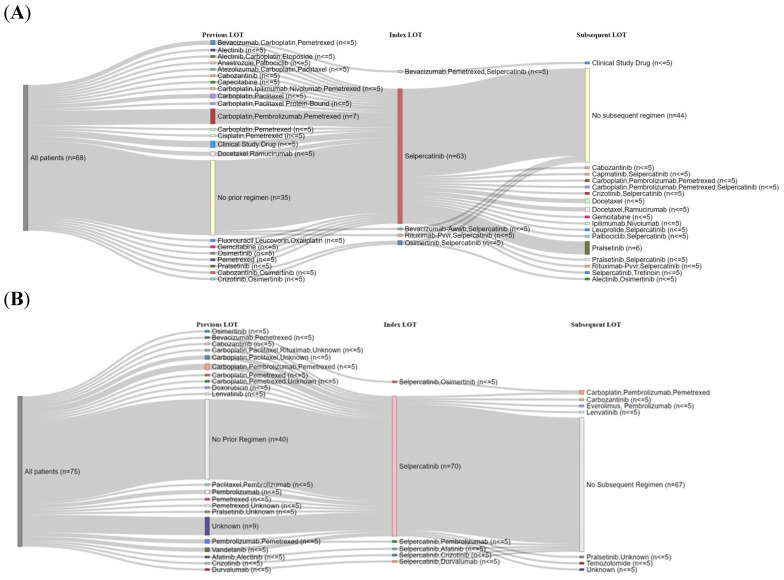

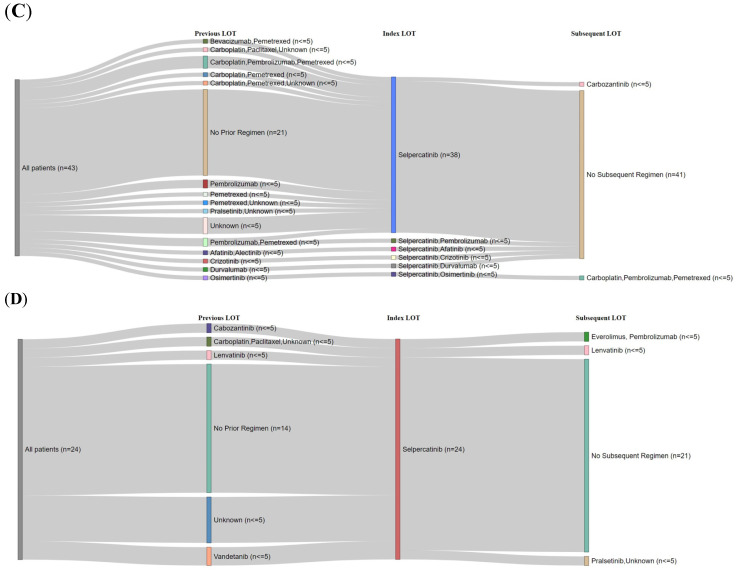
Sankey’s diagram: (**A**) FHD cohort; (**B**) CDM overall cohort; (**C**) CDM lung cancer cohort; and (**D**) CDM thyroid cancer cohort.

Of the FHD cohort, 35 (51.5%) patients did not receive prior anti-cancer treatment for metastatic disease, while 33 (48.5%) patients were previously treated. The majority (92.6%, n = 63/68) of patients initiated selpercatinib treatment as a monotherapy (Figure 2A). More than eighty percent of the patients received selpercatinib as a first-line (51.5%, n = 35) or second-line (32.4%, n = 22) treatment. Of the patients who received prior anti-cancer treatment, chemotherapy and chemo-immunotherapy were the most commonly used regimens (each used in 27.3%, n = 9/33), followed by tyrosine kinase inhibitors (18.2%, n = 6/33; including half of these patients who received EGFR tyrosine kinase inhibitors). Most patients (64.7%, n = 44) did not have subsequent treatment post selpercatinib. Of the patients who initiated a subsequent line of treatment (35.3%, n = 24), a third (33.3%, n = 8/24) continued to receive selpercatinib as a part of their new treatment regimen (Figure 2A). Of the 16 patients who did not continue with selpercatinib, the most common subsequent treatments were tyrosine kinase inhibitors (50.0%, n = 8/16).

Similar to the findings from FHD, the vast majority of patients (93.3%, n = 70/75) from the CDM began selpercatinib treatment as a monotherapy, including 88.4% of those with lung cancer and 100% of those with thyroid cancer (Figure 2B–D). Half (51.2%, n = 22/43) of the lung cancer patients and forty percent (41.7%, n = 10/24) of the thyroid cancer patients from the CDM cohort received prior anti-cancer treatment within 90 days prior to initiating selpercatinib. Similar to the FHD patients with *RET* fusion-positive a/mNSCLC, the most common treatments were chemotherapy (27.3%, n = 6/22) for the CDM lung cancer patients who received prior anti-cancer treatment.

### 3.4. Outcomes

**Figure 3:**
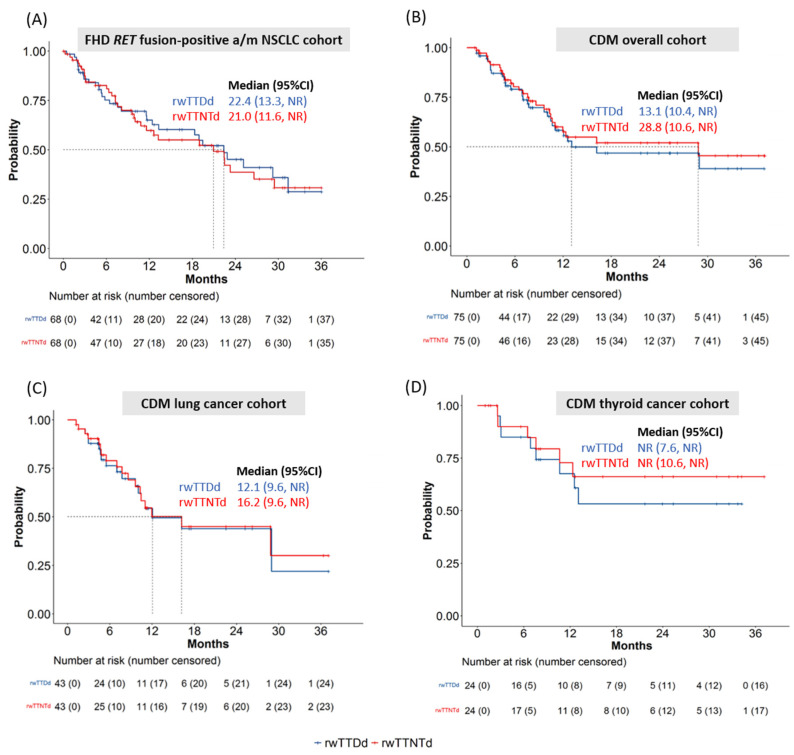
rwTTDd and rwTTNTd Kaplan–Meir curves from FHD and CDM cohorts: (**A**) FHD *RET* fusion-positive a/m NSCLC cohort; (**B**) CDM overall cohort; (**C**) CDM lung cancer cohort; and (**D**) CDM thyroid cancer cohort. Abbreviations, rwTTDd: real-world time to treatment discontinuation or death; rwTTNTd: real-world time to next treatment or death. Notes: rwTTDd was defined as the time from the index date to the last administration date of selpercatinib before an event (the initiation of a new LOT that did not include the selpercatinib, death, or a ≥ 90-day gap between the last selpercatinib administration and the last known activity date or the study end date, whichever occurred first) occurred. rwTTNTd was defined as the time from the index date to the start of the next line of treatment that may or may not include selpercatinib or death, whichever occurred first. If none of these conditions were met, patients were censored at the last known activity date or at the end of the study.

The median rwTTDd and rwTTNTd [95% confidence interval (CI)] for the FHD cohort (N = 68) were 22.4 [13.3–NR] and 21.0 [11.6–NR] months (Figure 3A). For the CDM cohort overall (N = 75), the median rwTTDd and rwTTNTd were 13.1 months [10.4–NR] and 28.8 months [10.6–NR], respectively (Figure 3B). In terms of specific cancer types within the CDM cohort, the lung cancer group (N = 43) had a median rwTTDd of 12.1 [9.6–NR] months and rwTTNTd of 16.2 [9.6–NR] months (Figure 3C). For the thyroid cancer group (N = 24), the median rwTTDd and rwTTNTd were not reached (Figure 3D).

### 3.5. Medication Adherence

The median MPR (IQR) was 0.95 (0.84–1.00) in overall CDM patients (N = 75), while it was 0.97 (0.85–1.00) in patients with lung cancer (n = 43) and 0.95 (0.83–1.00) in patients with thyroid cancer (n = 24). Furthermore, 77.3% patients in the overall CDM cohort, 81.4% patients in the lung cancer, and 75.0% patients in the thyroid cancer subgroups were considered adherent to selpercatinib treatment using the commonly used threshold of MPR ≥ 80%.

### 3.6. Sensitivity Analyses

Sensitivity analyses corresponding to rwTTDd and rwTTNTd resulted in naive medians or medians not reached (Figure S1), suggesting that the estimated medians may be sensitive to event/censoring definitions applied in this study. However, it may also imply that rwTTDd and rwTTNTd are statistically more powerful outcome measures by including a greater number of relevant events; thus, rwTTDd and rwTTNTd may provide more precise and better estimated medians. The MPR (with average MPR ≥ 0.8 across all groups) and proportion of patient adherence (over 70% across all groups) remained consistently high (Table S1).

## 4. Discussion

This descriptive study is the first to comprehensively report on the patient characteristics, treatment patterns, and associated outcomes among real-world patients treated with selpercatinib, including those with a/mNSCLC and thyroid cancer, in the US, expanding on our previously presented work [30]. Most patients in this study initiated selpercatinib as a monotherapy, consistent with label and guideline recommendations. Outcomes associated with the use of selpercatinib reported in this study directionally corroborate with known clinical trial results; however, a direct comparison is not advised given the differences in outcome definitions and lack of data on reasons for treatment discontinuation and other clinical features in the databases used in this study. Nonetheless, findings observed in this study demonstrate outcomes associated with use of selpercatinib in the real world, providing complementary evidence to that generated from clinical trials.

In patients with *RET* fusion-positive a/mNSCLC, the median rwTTDd and rwTTNTd estimates reported in this study from the FHD cohort are similar to previously reported median PFS, ranging from 22.0–24.9 months, in clinical trials [18,31]. The unreached rwTTDd and rwTTNTd among patients with thyroid cancer from the CDM cohort are consistent with the unreached median PFS reported in previous clinical trials [7,15]. Additionally, this study adds evidence on selpercatinib medication adherence that was not directly measured by previous clinical trials, suggesting that patients in the real world adhere well to selpercatinib treatment, with high MPR and adherence rates found across tumor types.

A few discrepancies in the outcomes are also noted. First, the median rwTTDd and rwTTNTd in patients with lung cancer from the CDM cohort appear to be shorter than the median PFS estimates reported from previous clinical trials. The differences, however, are plausible and could be due to differences in how these outcomes are defined; medians being immature; the uncertainty of *RET* alteration status and disease stage; the inclusion of small-cell lung cancer; and other different characteristics of the patient population not captured in the databases used in this study. Second, one sensitivity analysis showed a shorter median rwTTDd (12.6 months) relative to that observed in the main analysis in patients from the FHD cohort. This occurred because of differences in how outcomes were defined in the main versus the sensitivity analysis, where the rwTTDd estimation was confined to the current LOT regardless of selpercatinib use as part of the treatment in the next LOT. This sensitivity analysis did not account for the additional time on selpercatinib treatment of 33.3% patients that had a subsequent LOT continued with selpercatinib as part of their subsequent treatment regimen. As indicated by the LIBRETTO-001 study, patients may continue to benefit from selpercatinib therapy even after their disease has progressed [32]. Our observation, from the real-world data, supports that selpercatinib may offer sustained clinical advantage; however, the interpretation is limited here given the lack of progression data in the databases used in this study. Despite these discrepancies, the estimates were within the range of the PFS reported in studies conducted in real-world settings outside of the United States, which ranged from 11.9–16.9 months [19,20,21,22].

It is worth noting that compared to the patient population included in clinical trials, patients in our study cohort were relatively older (median age 67 years compared to the median age ranging from 60–63 years across trials) [18,31]. Our study cohort is also likely to be frailer, given the higher proportion of patients with a 2 or higher ECOG PS in the FHD (at least 11.8% vs. 2–5.8% across trials) [18,31] and over 20% of patients with CCI ≥ 3 in the CDM. These differences in patient characteristics at baseline may provide additional explanations for some of the observed discrepancies.

In the era of precision oncology, biomarker testing has emerged as a critical component. Our study underscores the importance of timely identification of *RET* alterations so that patients can receive an appropriate concordant therapy. Biomarker testing and guideline-concordant therapy are significantly associated with improved survival outcomes in patients with a/mNSCLC [33,34]. For patients with *RET* fusion-positive cancers, brain metastasis is common and responses to multikinase inhibitors are often suboptimal [35]. Therefore, clinicians should prioritize comprehensive genomic profiling, including next-generation sequencing (NGS) technology [20], to detect *RET* alterations as early as possible during the treatment trajectory. The integration of these tests into routine clinical practice can ensure the timely identification of patients with biomarker-driven disease and, consequently, the appropriate initiation of biomarker-targeted treatments (such as *RET* inhibitors). This approach optimizes the therapeutic benefit upfront, especially in patients with advanced disease.

One of the key strengths of this study is that, despite the rarity of *RET* alterations making it challenging to study patients with such alterations in real-world databases, this study was able to present findings from two data sources across tumor types. This study provides key insights into the real-world outcomes of selpercatinib in the United States, which is largely consistent with outcomes reported in clinical trials. This study also has several limitations. The generalizability of study findings is limited, as the databases used in this study are not entirely representative of all cancer patients in the US. The retrospective and descriptive study design may introduce certain biases and/or limit our interpretation of findings due to missing information and incomplete follow-ups. Specifically, key disease characteristics were not available in the CDM claims data, while data on reasons for discontinuation and disease progression status were not available in both the databases used in this study. We could not confirm *RET* positivity status in the CDM cohort given the lack of this information in a claims-only database; however, we required patients to have at least two prescriptions of selpercatinib during the post-index period, assuming that only patients with a confirmed *RET* alteration status would receive a second fill. The results must be interpreted with caution given the wide 95% CIs resulting from the limited sample size, which reflects the rarity of *RET*-altered cancers.

Several areas for future research are suggested. As more patients with *RET*-altered cancers are tested and identified in the real world, studies with a larger sample size are needed to confirm our findings, as well as CNS outcomes related to the real-world use of selpercatinib. Subgroup analyses in patients with other *RET* fusion-positive tumors other than lung or thyroid cancers are necessary to provide real-world evidence of selpercatinib in other populations observed in clinical trials [36]. Lastly, future investigations can focus on identifying factors associated with a positive treatment response, aiding in the early identification of responders versus non-responders.

## 5. Conclusions

Selpercatinib-treated patients in the real world were more heterogenous than those typically included in clinical trials. Observed real-world outcomes associated with selpercatinib use in a cohort of older and frailer patients were largely consistent with those reported in clinical trials. These findings provide complimentary evidence to that generated from clinical trials and continue to support the use of selpercatinib as the standard of care for patients with *RET*-altered cancers in a real-world setting, including those with lung and thyroid cancers. These results also underscore the importance of early testing to confirm the presence or absence of *RET* alterations to ensure that all patients with *RET*-altered cancers can benefit from receiving appropriate *RET*-targeted treatment.

## Figures and Tables

**Table 1:** Patient demographics and clinical characteristics at baseline for patients in FHD.

	FHD, Overall a/m NSCLC (N = 68)
Demographics	
Age at index date (year), median (IQR) ^a^	67.0 (60.0–74.0)
Age 65+ at index date (year), n (%)	40 (58.8)
Male, n (%)	38 (55.9)
Race, n (%) ^b^	
White	45 (66.2)
Non-white	9 (13.2)
Geographic region, n (%) ^b^	
Midwest	n ≤ 5
Northeast	8 (11.8)
South	16 (23.5)
West	7 (10.3)
Payer type, n (%) ^c^	
Commercial Health Plan	47 (69.1)
Medicare	9 (13.2)
Other (including Medicaid, self-pay etc.)	12 (17.6)
Clinical characteristics	
Non-smokers, n (%)	38 (55.9)
Body weight(kilogram), mean (SD) ^d^	82.5 (23.1)
Practice type, n (%)	
Academic	25 (36.8)
Community	43 (63.2)
Stage at initial diagnosis, n (%) ^b^	
Stage IB-IIIC	11 (16.1)
Stage IV	56 (82.4)
Non-squamous cell carcinoma Histology, n (%)	67 (98.5)
ECOG PS at index date, n (%) ^b^	
0	22 (32.4)
1	28 (41.2)
2	8 (11.8)
PD-L1 positive ^e^	43 (63.2)
Follow-up time, median (IQR)	13.1 (6.6–26.8)

Abbreviations: a/mNSCLC, advanced/metastatic non-small cell lung cancer; ECOG PS, Eastern Cooperative Oncology Group Performance Status; IQR, interquartile range; PD-L1, programmed death-ligand 1; SD, standard deviation. Notes: ^a^ Patients with a birth year of [Data Cutoff Year—85] or earlier may have an adjusted birth year in Flatiron Health datasets due to patient de-identification requirements. ^b^ Counts and percentages may not add up to 100% due to missing or rounding off of decimal places. ^c^ The other payer type included patient assistance program, self-pay, other unknown payer types and missing. ^d^ BMI was identified within 30 days prior or up to 15 days after the patient’s index date. ^e^ PD-L1 tumor expression of ≥1%.

**Table 2:** Patient demographics and clinical characteristics at baseline for patients in CDM.

	CDM, Overall (N = 75)
Demographics	
Age at index date (year), median (IQR)	65.0 (55.0–74.0)
Age 65+ at index date (year), n (%)	41 (54.7)
Male, n (%)	41 (54.7)
Race, n (%) ^a^	
White	51 (68.0)
Non-white	22 (29.3)
Geographic region, n (%) ^a^	
Midwest	18.0 (24.0)
Northeast	7.0 (9.3)
South	25.0 (33.3)
West	25.0 (33.3)
Payer type, n (%)	
Commercial Health Plan	41.0 (54.7)
Medicare	34.0 (45.3)
Clinical characteristics	
Charlson Comorbidity Index ^b^	
0	26 (34.7)
1–2	33 (44.0)
≥3	16 (21.3)
Presence of Baseline Metastasis, n (%)	48 (64.0)
Site of Metastasis, n (%) ^c^	
Bone/BM	15 (31.3)
Brain/CNS	9 (18.8)
Liver	10 (20.8)
Lung	11 (22.9)
Lymph	14 (29.2)
Pleura	7 (14.6)
Follow-up time, median (IQR)	8.6 (4.8–16.7)

Abbreviations: IQR, interquartile range; SD, standard deviation. Notes: ^a^ Counts and percentages may not add up to 100% due to missing or rounding off of decimal places. ^b^ Excluding cancers. ^c^ Patients could have more than one site of metastasis.

## Data Availability

Data from the Flatiron Health database that support the findings of this study were originated by, and are the property of, Flatiron Health, Inc., which has restrictions prohibiting the authors from making the dataset publicly available. Requests for data sharing by license or by permission for the specific purpose of replicating results in this manuscript can be submitted to PublicationsDataAccess@flatiron.com (accessed on 22 June 2022). Another part of the data presented in this study have been originated by Optum’s de-identified Clinformatics^®^ Data Mart Database.

## Supplementary Materials

The following supporting information can be downloaded at: https://www.mdpi.com/article/10.3390/cancers16223835/s1, Table S1: Sensitivity analysis of medication adherence; Figure S1: Results of sensitivity analyses for TTDd, TTD and TTNT Kaplan–Meir curves from FHD and CDM cohorts.
