# Supplementary material for: Real-World Outcomes of Selective RET Inhibitor Selpercatinib in the United States: Descriptive, Retrospective Findings from Two Databases
Source: Cancers (Basel). 2024 Nov 15;16(22):3835. doi: 10.3390/cancers16223835 (PMC11592841; doi:10.3390/cancers16223835)
Supplement: Supplementary file 1 [file cancers-16-03835-s001.zip › cancers-3256791-supplementary.pdf]

Supplementary Materials

Table S1. Sensitivity analysis of medication adherence

	CDM, overall	CDM, lung cancer	CDM, thyroid cancer
Patients with 2 fills during 180 days of follow-up	51	27	18
MPR (in 180 days), median (IQR)	0.93 (0.69-1.00)	0.97 (0.77-1.00)	0.92 (0.67-0.97)
Patient adherent (in 180 days), n(%)	36 (70.6)	20 (74.1)	13 (72.2)

Notes: the sensitivity analysis of MPR limited the calculation within those who had at least 180 days of follow-up.

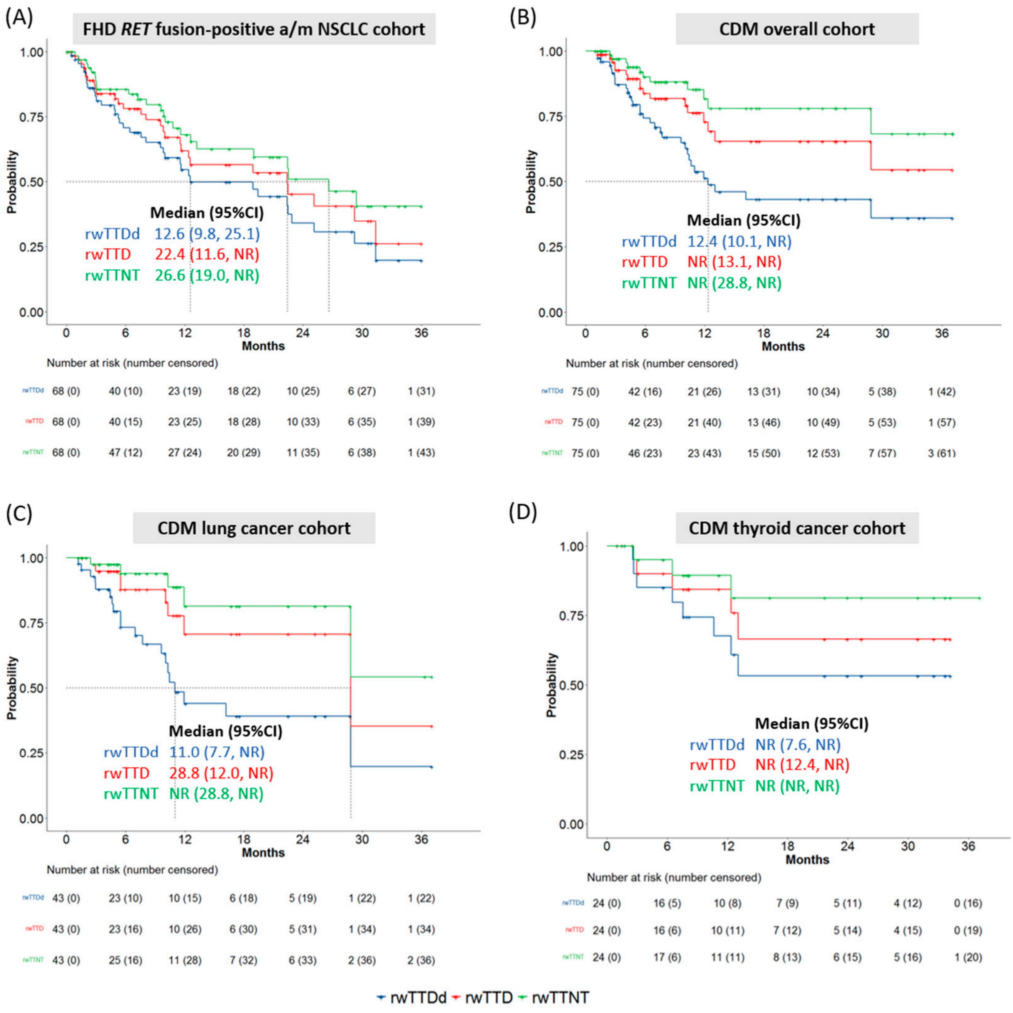

Figure S1. Results of sensitivity analyses for rwTTDd, rwTTD and rwTTNT Kaplan-Meir curves from FHD and CDM cohorts. (A) FHD *RET* fusion-positive a/m NSCLC cohort. (B) CDM overall cohort. (C) CDM lung cancer cohort. (D) CDM thyroid cancer cohort.
Abbreviations: rwTTDd, real-world time to treatment discontinuation or death; rwTTNT, real-world time to next treatment; rwTTNTd, real-world time to next treatment or death. Notes: the rwTTDd (sensitivity) considered initiation of new line of treatment as treatment discontinuation, regardless of whether the new treatment included selpercatinib or not. The rwTTD (sensitivity) and rwTTNT (sensitivity) considered death as censoring rather than an event.
